# Competitive Interactions between Parasitoids Provide New Insight into Host Suppression

**DOI:** 10.1371/journal.pone.0082003

**Published:** 2013-11-28

**Authors:** Hai-Yun Xu, Nian-Wan Yang, Fang-Hao Wan

**Affiliations:** State Key Laboratory for Biology of Plant Diseases and Insect Pests, Institute of Plant Protection, Chinese Academy of Agricultural Sciences, Beijing, P. R. China; The Evergreen State College, United States of America

## Abstract

Understanding the dynamics of potential inter- and intraspecific competition in parasitoid communities is crucial in the screening of efficient parasitoid species and for utilization of the best parasitoid species combinations. In this respect, the host-parasitoid systems, *Bemisia tabaci* and two parasitoids, *Eretmocerus hayati* (exotic) and *Encarsia sophia* (existing) were studied under laboratory conditions to investigate whether interference competition between the exotic and existing species occurs as well as the influence of potential interference competition on the suppression of the host *B. tabaci*. Studies on interspecific-, intraspecific- and self-interference competition in two parasitoid species were conducted under both rich and limited host resource conditions. Results showed that (1) both parasitoid species negatively affect the progeny production of the other under both rich and limited host resource conditions; (2) both parasitoid species interfered intraspecifically on conspecific parasitized hosts when the available hosts are scarce and; 3) the mortality of *B. tabaci* induced by parasitoids via parasitism, host-feeding or both parasitism and host-feeding together varied among treatments under different host resource conditions, but showed promise for optimizing control strategies. As a result of our current findings, we suggest a need to investigate the interactions between the two parasitoids on continuous generations.

## Introduction

In nature, a single species of host can have multiple species of natural enemies [1-4], which often results in intense interactive competition/interference for the host resource [5]. Competitive interaction frequently occurs in parasitoids, because parasitoids, unlike most predators, usually have narrow host ranges [5]. Godfray [6] emphasized that competition in parasitoid communities is a key factor in shaping the structure of natural enemy communities. Understanding the interspecific competition among parasitoid species is crucial to the selection of appropriate biological control agents for introduction and release [7]. 

Consequently, two crucial factors have to be considered before importing and using the exotic parasitoid species in a biological control system: 1) does the exotic species compete with existing parasitoid populations for the shared hosts and 2) what is the impact of multiple parasitoids on the suppression of a shared host? Studies are needed to investigate the dynamics of competitive interactions among parasitoids and how competition could influence pest suppression [8]. Unfortunately, there is little information on interspecific competition and the effect of these competitive interactions on community structure and dynamics [3,6,9-12].

Competition among parasitoid species might be direct or indirect [13]. Lethal interference competition, which is the crucial interaction between parasitoids, refers to the direct interaction that leads to the death of the competitor [14]. This phenomenon commonly occurs among solitary parasitoids and often plays an important role in determining the structure of the insect community, and is of growing concern [8,12,15-17]. Results of the studies addressing this issue suggest a need to consider mechanisms of lethal interference competition in theoretical and empirical research on parasitoid competition in order to better understand coexistence and host suppression in biological control practice [8,16-21]. 

Here we investigated the competitive interactions between *Encarsia sophia* (Girault & Dodd) and *Eretmocerus hayati* (Zolnerowich & Rose) (Hymenoptera: Aphelinidae), two key parasitoids of the whitefly *Bemisia tabaci* (Gennadius) (Hemiptera: Aleyrodidae) Middle East-Asia Minor 1 (MEAM1, also called biotype B) [22], a serious pest in vegetables and broad-acre crops worldwide [23-25]. *B. tabaci* MEAM1 is one of the most serious pests throughout China. Since its first invasion in China in the mid-1990s, it had spread into most provinces of China, resulting in serious economic losses [26-28]. *En. sophia* (formerly known as *En. transvena*) is a solitary, arrhenotokous, heteronomous autoparasitoid. In this species, fertilized eggs (producing females) are laid in whitefly nymphs and the unfertilized eggs (producing males) are laid externally on immature parasitoids inside the whitefly host, either on conspecific species or on heterospecific primary parasitoids [18,29,30]. *En. sophia*, which originated in Pakistan, is currently present across northern and southern China and has proved to be a promising parasitoid of *B. tabaci* [19,31,32]. *Er. hayati* is a newly imported parasitoid species, also from Pakistan, but is still under evaluation in China [33,34]. It is a primary, solitary parasitoid which oviposits externally under the nymphal host [35]. After eclosion, the first instar larva penetrates the host from underneath and develops internally [33,35]. *Er. hayati* has caused substantial reductions of *B. tabaci* abundance after its introduction into the USA [36-38]. Based on the CLIMEX model indices and observations on establishment in USA, *Er. hayati* offered the best prospects for introduction in Australia and China [39]. Given this background, *Er. hayati* was introduced into quarantine at the Institute of Plant Protection, Chinese Academy of Agricultural Sciences (CAAS), Beijing, China from Texas in 2008 by scientists from the State Key Laboratory for Biology of Plant Diseases and Insect Pests. In our previous studies, we have proved that *Er. hayati* is a good candidate for biological control of *B. tabaci* MEAM1 in China [33].

Both of these parasitoids attack all nymphal stages (N1-N4) of *B. tabaci* [33,34], so interspecific competition is likely. The objective of the present study was to determine whether the introduction of an exotic parasitoid would result in interference between the existing parasitoid, thus affecting the suppression of *B. tabaci*. Interspecific-, intraspecific- and self-interference competition experiments were conducted under laboratory conditions to investigate whether interference competition between these two species occurs. Availability of hosts is critical for parasitoid reproduction [40,41]. Therefore, the experiments were conducted under two levels of host resource conditions: rich (30 hosts available) and limited (10 hosts available) to better understand the relationship between host availability and the intensity of competition. 

## Materials and Methods

### Study organisms

The laboratory colony of *B. tabaci* MEAM1 was collected from greenhouses at the Institute of Vegetables and Flowers, Chinese Academy of Agricultural Sciences (CAAS) in Beijing, and has been maintained under glasshouse conditions without exposure to insecticides for 4 years. The laboratory colony of *En. sophia* was provided by the Institute of Plant and Environment Protection, Beijing Academy of Agriculture and Forestry Sciences. The laboratory colony of *Er. hayati* was provided by the Vegetable Integrated Pest Management Laboratory, Texas Agricultural Experiment Station at Weslaco, TX, USA. The laboratory colonies of these parasitoids were established using *B. tabaci* MEAM1as the host insect and tomato, *Solanum lycopersicum* L. var. *lycopersicum* (Solanaceae) plants (cv. Zhongyan 988, Zhongyanyinong Seed Technology Co. Ltd., Beijing, China), as host plants in the laboratory experiments. 

All host plants and insect colonies were kept at 26°C ± 2°C, 65 ± 5 % RH, and 14L: 10D regime, at Langfang Experimental Station (39°30’ N, 116°36’ E), Langfang, Hebei Province, China.

### Experimental design and implementation

Tomato plants were grown individually in plastic pots (13cm diameter, 11cm height) in an air conditioned greenhouse without insect contamination. Plants that were 15 cm tall, with 5 - 7 expanded leaves were used in the experiments. All leaves except one top true leaf were removed 2 days before use. The plants were moved to the laboratory and exposed for 24 h to 20 - 50 adult *B. tabaci* MEAM1 per plant for egg laying, then all adults were removed. After the exposure, the plants were transferred to cages with fine-mesh nylon screen (pore diameter 0.125mm) to preventing further whitefly contamination. All experiments were conducted and maintained under 26 ± 2 °C and 14:10 L: D regime.

After 14 - 15 days, a round clip cage (23 mm diameter) was affixed to each leaf. It is known that each female of *Er. hayati* or *En. sophia* consumes (both parasitizing and host-feeding) less than 20 hosts per day [34]. For this reason, all but 30 (rich host resource condition) or 10 (limited host resource condition) *B. tabaci* MEAM1 nymphs (second-third instar) were removed from the area inside each leaf cage. 

For experiments, newly emerged (< 12h old) parasitoids were mated (one female with one male) and kept for 24 h in a 1.5-ml microcentrifuge tube with a cotton thread saturated with 5% honey: water solution. All experimental females were observed mating. Four kinds of interference treatments were conducted under each host resource condition:

1. Interspecific interference treatment. One female was introduced into the clip cage for 24 h and removed immediately followed by the introduction of a heterospecific female into the same clip cage for another 24 h (‘S/H’, *En. sophia* female introduced first, and *Er. hayati* female introduced subsequently; ‘H/S’, *Er. hayati* female introduced first, and *En. sophia* female introduced subsequently).
2. Intraspecific interference treatment. After the first female was removed, a conspecific female was introduced for 24 h (‘S1/S2’, after the removal of first *En. sophia* female, another *En. sophia* female introduced subsequently; ‘H1/H2’, after the removal of first *Er. hayati* female, another *Er. hayati* female introduced subsequently).
3. Self-interference treatment. The female was checked after the first 24 h, then it was kept in the same clip leaf cage for another 24 h (‘S1/S1’, the same *En. sophia* female was kept in the clip leaf cage for 48h; ‘H1/H1’, the same *Er. hayati* female was kept in the clip leaf cage for 48h);
4. Alone treatment. The first female was removed after 24 h without further introduction into the clip leaf cage, i.e., no subsequent competitor (‘S’, *En. sophia* female was kept in the clip leaf cage for 24h; ‘H’, *Er. hayati* female was kept in the clip leaf cage for 24h).

Treatments were assigned randomly. After all the parasitoids were removed, the plants were kept in isolation for 10 - 14 additional days. The number of parasitoid pupae, dead whiteflies, and unparasitized whiteflies (whitefly exuviae) on the leaves were recorded. “Dead” whiteflies had a flattened, dried, and discoloured appearance [16]. 

### Data analyses

Since the data did not fit normal distribution even after transformation, non-parametric analyses were conducted (SPSS version 19.0 software package). Difference of means of progeny production of both parasitoid species, as well as the mean number of dead hosts between the alone treatment and the competition treatments were compared with Mann-Whitney test. Difference of the number of dead hosts induced by parasitism, host-feeding, or by both parasitism and host-feeding among different treatments were compared with Kruskal-Wallis test. Multiple comparisons after the Kruskal-Wallis test were performed using the Tukey test. Difference of the mortality of *B. tabaci* between the two orders that *B. tabaci* MEAM1 infested leaf exposed to parasitoids (S/H or H/S) were compared with Mann-Whitney test. The levels of significance were set at *P* < 0.05.

Mechanisms of inter- and intraspecific interference competition under limited resource conditions were inferred by two additional analyses that followed Collier & Hunter’s [16] methods. 

## Results

### The outcome of interference competition between parasitoids

#### Interspecific interference competition

**Figure 1:**
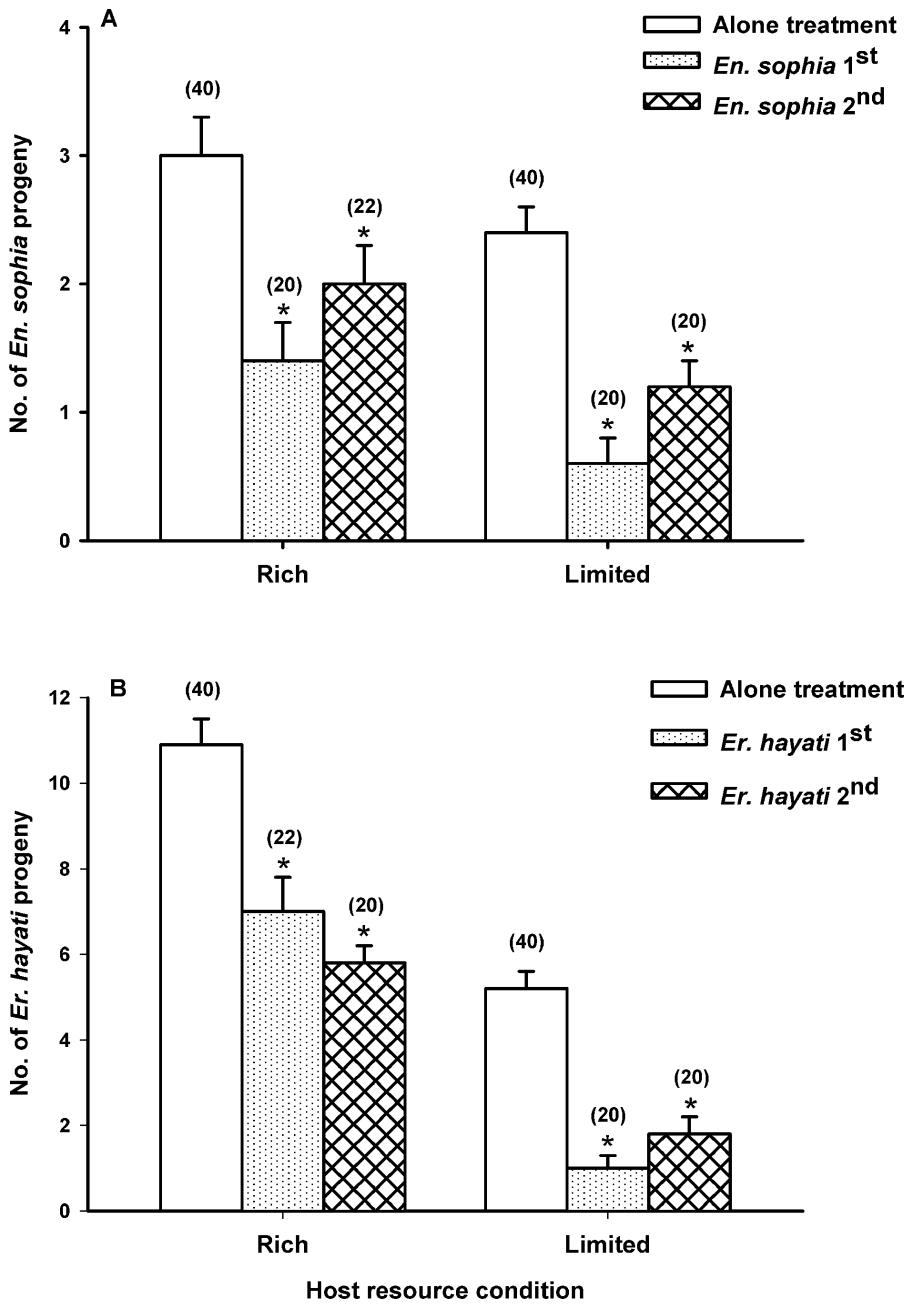
Interspecific interference competition effect on parasitoid progeny production. The blank, punctate and netted bars represent the number (mean ± SE) of *En*. *sophia* (A) or Er. *hayati* (B) progeny when introduced into the leaf cage alone (no subsequent parasitoid), first [introduced before a Er. *hayati* (A) or *En*. *sophia* (B) female] or second [introduced after a Er. *hayati* (A) or *En*. *sophia* (B) female], respectively. Note: * above each bar indicates the number of progeny that differed significantly between alone treatment and both the interference treatments, respectively (Mann-Whitney test, *p* < 0.05). Numbers in parentheses above bars indicate the sample sizes of treatments.

The subsequent introduction of heterospecific females reduced the progeny of first introduced females under both rich and limited host resource conditions in the interspecific interference treatment (Figure 1). As compared to the *En. sophia* alone treatment, the number of *En. sophia* progeny was reduced significantly (1.6 and 1.8 offspring) by the subsequent introduction of *Er. hayati* under both rich and limited resource conditions (Figure 1A; *Mann-Whitney U* = 209.00 and 86.50, *P* = 0.002 and < 0.0001, respectively). Likewise, the number of *Er. hayati* progeny was reduced significantly (3.9 and 4.2 offspring) by the subsequent introduction of *En. sophia* under both rich and limited resource conditions (Figure 1B; *Mann-Whitney U* = 199.00 and 53.50, *P* = 0.0004 and < 0.0001, respectively). 

The order of introduction affected the strength of interspecific interference which was represented by the number of parasitoid progeny. In most cases, progeny production of parasitoid was less affected by interference competition when it was introduced second into the leaf cage than when it was introduced first. When *En. sophia* was introduced after *Er. hayati* into the leaf cage, the numbers of *En. sophia* progeny were reduced relative to the alone treatment by 33 and 50% under the rich host resource condition and limited host resource condition (Figure 1A; *Mann-Whitney U* = 313.50 and 212.00, *P* = 0.056 and 0.002, respectively), while the progeny was reduced by 53 and 75% when *En. sophia* was introduced before *Er. hayati* under corresponding resource conditions (Figure 1A; *Mann-Whitney U* = 209.00 and 86.50, *P* = 0.0022 and < 0.0001). When *Er. hayati* was introduced after *En. sophia* into the leaf cage, the number of *Er. hayati* progeny was reduced by 65% of that in the alone treatment under the limited host resource condition (Figure 1B; *Mann-Whitney U* = 102.00, *P* < 0.0001), while progeny was reduced by 81% when it was introduced before *En. sophia* (Figure 1B; *Mann-Whitney U* = 53.50, *P* < 0.0001). However, the relationship was reversed for *Er. hayati* under the rich host resource condition: when *Er. hayati* was introduced after *En. sophia* into the leaf cage, the number of *Er. hayati* progeny was reduced by 47% of that in the alone treatment (Figure 1B; *Mann-Whitney U* = 78.50, *P* < 0.0001), but 27% when it was introduced before *En. sophia* (Figure 1B; *Mann-Whitney U* = 199.00, *P* = 0.0004).

Host resource richness affected intensity and outcome of interspecific competition. Under the rich host resource condition, *Er. hayati* produced the greater proportion of progeny regardless of the order of introduction (introduced before *En. sophia*: 7.0/(7.0+2.0)=0.78, introduced after *En. sophia*: 5.8/(5.8+1.4)=0.81) in the competition treatment. Under the limited host resource condition, the second-introduced female produced the greater proportion of progeny regardless of the parasitoid species (*En.sophia* introduced after *Er. hayati*: 1.2/(1.2+1)=0.55, *Er. hayati* introduced after *En. sophia*: 1.8/(0.6+1.8)=0.75). 

#### Intraspecific interference competition

For intraspecific interference competition, the progeny of the first and second introduced female were unable to be distinguished as in interspecific interference treatments. For *En. sophia*, under the rich host resource condition, when compared with the alone treatment, the total number of progeny slightly increased in self- (Table 1; *Mann-Whitney U* = 232.00, *P* = 0.004) and conspecific interference competition treatments (Table 1; *Mann-Whitney U* = 309.50, *P* = 0.15). However, the total number of progeny in either competition treatment did not double as expected compared to the alone treatment. Under the limited host resource condition, the total number of progeny of *En. sophia* in self- and conspecific competition decreased when compared with that in alone treatment, but the differences were not significant (Table 1; *Mann-Whitney U.* = 353.00 and 295.00, *P.* = 0.45 and 0.087 in self- and conspecific interference competition treatment, respectively).

**Table 1:** Mean number (± SE) of progeny of *Encarsia sophia* or *Eretmocerus hayati* in alone, self- and conspecific interference treatments under the rich and limited host resource conditions.

Parasitoid species	Parasitoids progeny					
	Under the rich host resource condition			Under the limited host resource condition		
	Alone	Self	Conspecific	Alone	Self	Conspecific
*En. sophia*	3.0 ± 0.3	5.1 ± 0.7*	4.0 ± 0.6	2.4 ± 0.2	2.1 ± 0.4	1.8 ± 0.3
	(40)	(21)	(20)	(40)	(20)	(20)
*Er. hayati*	10.9 ± 0.6	11.3 ± 0.8	12.5 ± 1.2	5.2 ± 0.4	4.6 ± 0.5	3.4 ± 0.3*
	(40)	(30)	(22)	(40)	(20)	(20)

Note: * indicate the parasitoid mean progeny in self- or conspecific interference treatment significantly differed as compared to that in alone treatment (Mann-Whitney test, *p < 0.05*). Numbers in parentheses indicate the sample sizes of treatments.

For *Er. hayati*, under the rich host resource condition, the total number of progeny did not vary significantly in self- or conspecific interference competition treatments, as compared with that in the alone treatment (Table 1; *Mann-Whitney U* = 535.00 and 366.50, *P.* = 0.44 and 0.28 in self- and conspecific interference competition treatment, respectively). Under the limited host resource condition, the total number of progeny decreased significantly in the conspecific interference competition treatment as compared to that in the alone treatment (Table 1; *Mann-Whitney U* = 191.00, *P* = 0.001), while the decrease in self-interference competition treatment was not significant (Table 1; *Mann-Whitney U* = 323.50, *P* = 0.22).

### Mortality of the host *B. tabaci* MEAM1 caused by parasitoids

#### *En. sophia* introduced first

**Figure 2:**
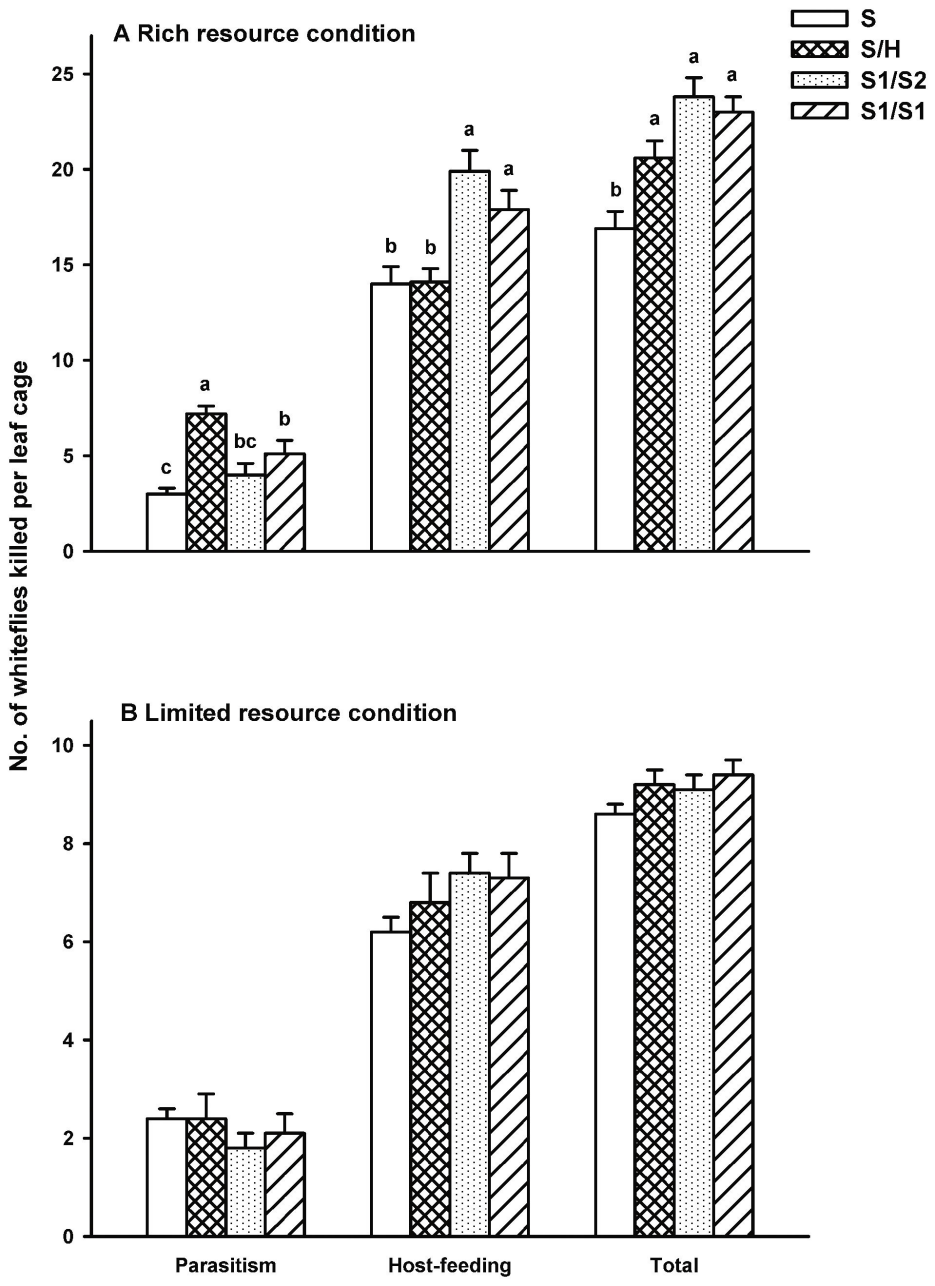
Mean number of whitefly killed by parasitoids via parasitism, host-feeding, and both parasitism and host-feeding when *Encarsia* *sophia* was introduced into the leaf cage first. A: under the rich host resource condition; B: under the limited host resource condition. An area of 3.5 cm^2^ of a leaf on a potted tomato plant was covered by a clip cage. S, S/H, S1/S2 and S1/S1 represent the four treatments: *En. sophia* female was introduced into the leaf cage alone for 24h (with no subsequent parasitoid female introduced), followed by a heterospecific female (*Er. hayati*) for another 24 h, followed by a conspecific female (*En. sophia*) for another 24 h and followed by itself for another 24h, respectively. Sample sizes of treatments S, S/H, S1/S2 and S1/S1 are 40, 20, 20, 21 and 40, 20, 20, 20 under the rich and limited host resource condition, respectively. Bar heads with different lowercase letters in each cluster indicate significant differences in number of hosts killed among different treatments (multiple comparison procedure based on the Tukey test after the Kruskal-Wallis test, *P < 0.05*); no significant difference was found under the limited resource condition (Kruskal-Wallis test, χ^2^ = 2.63, 6.11, and 7.67, *df* = 3, *P* = 0.45, 0.11, and 0.053).

Under the rich host resource condition, parasitism in interspecific interference treatment S/H was significantly greater than other treatments (Figure 2A; χ^2^ = 32.79, *df* = 3, *P* < 0.0001). The highest number of the hosts killed by parasitoids via host-feeding was found in the intraspecific interference treatment S1/S2, followed by the self-interference treatment S1/S1, while the subsequent introduction of *Er. hayati* (interspecific interference treatment S/H) did not increase the number of hosts fed as compared to *En. sophia* alone (Figure 2A; χ^2^ = 21.23, *df* = 3, *P* < 0.0001). The total number of the hosts killed by parasitoids via both parasitism and host-feeding differed significantly among treatments. The subsequent introduction of a second female (heterospecific, conspecific or same) onto the leaf that had previously been exposed to one *En. sophia* female increased the total mortality of hosts as compared to the *En. sophia* alone treatment (Figure 2 A; χ^2^ = 27.14, *df* = 3, *P* < 0.0001). 

Under limited host resource condition, no significant difference in parasitism was seen among treatments (Figure 2B; χ^2^ = 2.63, *df* = 3, *P* = 0.45). The numbers of hosts killed by parasitoids via host-feeding among treatments were similar (Figure 2B; χ^2^ = 6.11, *df* = 3, *P* = 0.11). In addition, the total number of hosts killed by parasitoids via both parasitism and host-feeding did not vary significantly among treatments (Figure 2B; χ^2^ = 7.67, *df* = 3, *P* = 0.053).

#### *Er. hayati* introduced first

**Figure 3:**
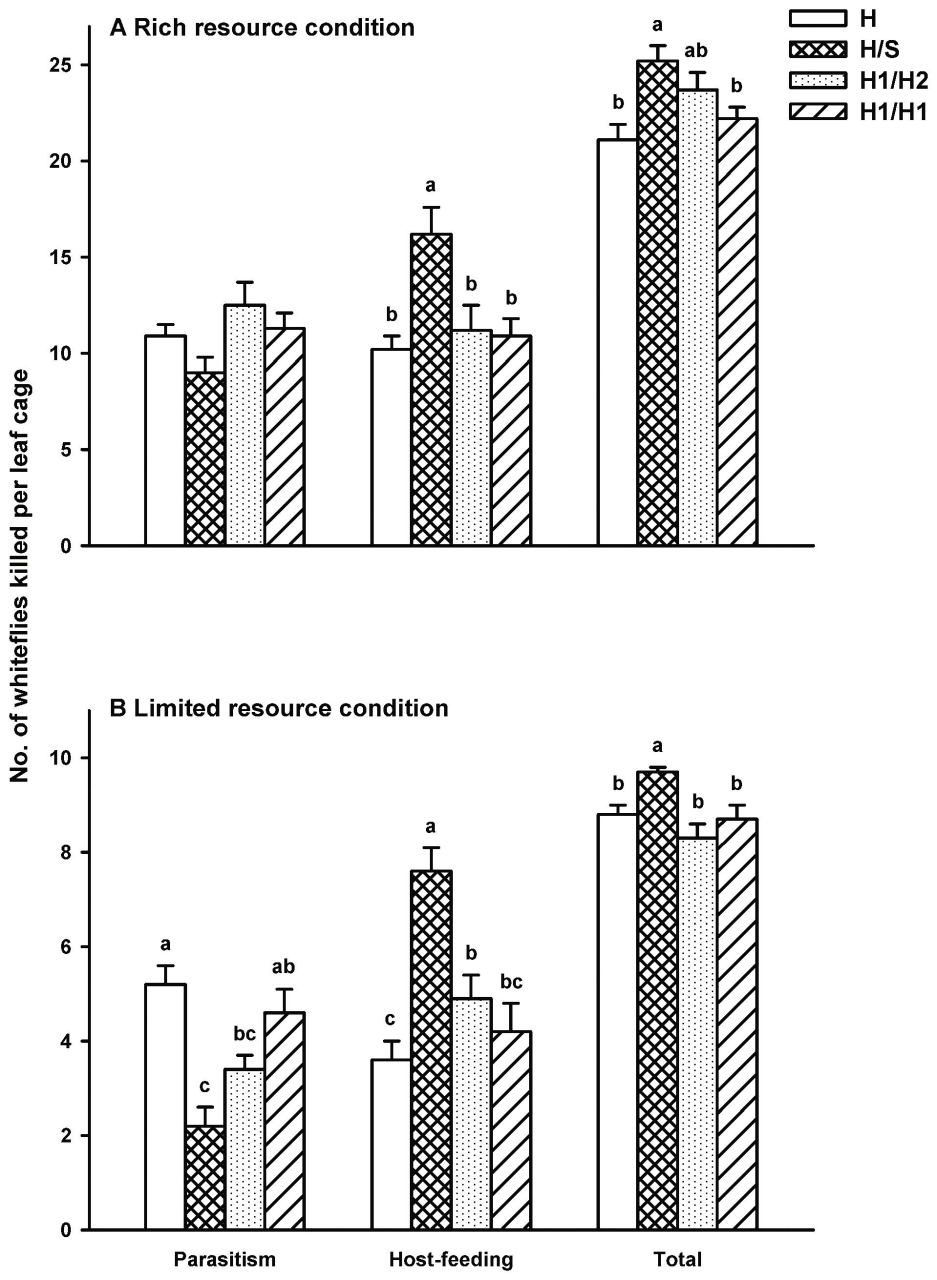
Mean number of whitefly killed by parasitoids via parasitism, host-feeding, and both parasitism and host-feeding when *Eretmocerus* *hayati* was introduced into the leaf cage first. A: under the rich host resource condition; B: under the limited host resource condition. An area of 3.5 cm^2^ of a leaf on a potted tomato plant was covered by a clip cage. H, H/S, H1/H2 and H1/H1 represented the four treatments: *Er. hayati* was introduced into the leaf cage alone for 24 h (with no subsequent parasitoid female introduced), followed by a heterospecific female (*En. sophia*) for another 24 h, followed by a conspecific female (*Er. hayati*) for another 24 h and followed by itself for another 24h, respectively. Sample sizes of treatments H, H/S, H1/H2 and H1/H1 are 40, 22, 22, 30 and 40, 20, 20, 20 under the rich and limited host resource condition, respectively. Bar heads with different lowercase letters in each cluster indicate significant differences in number of hosts killed among different treatments (multiple comparison procedure based on the Tukey test after the Kruskal-Wallis test, *P < 0.05*); no significant difference was found in parasitism under the rich host resource condition (Kruskal-Wallis test, χ^2^ = 6.92, *df* = 3, *P* = 0.074).

Regardless of the rich or limited host resource conditions, the subsequent introduction of the heterospcific parasitoid *En. sophia* (H/S treatment) caused the lowest number of total progeny among treatments, although it was not significantly different under the rich host resource condition (Figure 3A; χ^2^ = 6.92, *df* = 3, *P* =0.074) but significantly different under the limited host resource condition (Figure 3B; χ^2^ = 28.46, *df* = 3, *P* < 0.0001).

Under the rich host resource condition, the highest number of host killed by parasitoids via host-feeding was found in the interspecific interference treatment H/S (Figure 3A; χ^2^ = 13.17, *df* = 3, *P* = 0.004). The total number of the hosts killed by parasitoids via both parasitism and host-feeding differed significantly among treatments (Figure 3A). The subsequent introduction of one *En. sophia* female onto the leaf that had previously been exposed to one *Er. hayati* female (H/S treatment) caused the highest mortality of host (Figure 3A; χ^2^ = 14.88, *df* = 3, *P* = 0.002). 

Under the limited host resource condition, the highest number of the host killed by parasitoids via host-feeding was found in the interspecific interference treatment H/S, followed by the conspecific interference treatment H1/H2 and self-interference treatment H1/H1 (Figure 3B; χ^2^ = 31.48, *df* = 3, *P* < 0.0001). The total number of the hosts killed by parasitoids via both parasitism and host-feeding differed significantly among treatments (Figure 3B). The highest total mortality of host was found in the interspecific interference treatment H/S (Figure 3B; χ^2^ = 14.06, *df* = 3, *P* = 0.003).

When focused on the interspecific interference competition between these two parasitoids, the order of infested leaf exposed to parasitoids (S/H vs. H/S) did not affect the mortality of the host *B. tabaci* MEAM1 induced by parasitoids via parasitism (limited: *Mann-Whitney U* = 197.50, *P* = 0.95; rich: *Mann-Whitney U* = 146.00, *P* = 0.060), host-feeding (limited: *Mann-Whitney U* = 167.00, *P* = 0.37; rich: *Mann-Whitney U* = 166.00, *P* = 0.17) and both parasitism and host-feeding (limited: *Mann-Whitney U* = 151.00, *P* = 0.12) in most cases. However, when an *Er. hayati* female was introduced first and *En. sophia* was introduced subsequently under the rich host resource condition, host mortality caused by both parasitism and host-feeding was higher than that in the reverse situation (*Mann-Whitney U* = 95.00, *P* = 0.002).

### Mechanisms of lethal interference competition

#### Interspecific interference

As compared to the *En. sophia* alone treatment (S), the subsequent introduction of *Er. hayati* (S/H) did not significantly increase the number of dead hosts (by 0.6 = 6.8-6.2; Table 2; *Mann-Whitney U* = 335.00, *P* = 0.30), indicating that host-feeding on *En. sophia* parasitized hosts was not the way that *Er. hayati* interfered with *En. sophia*. However, the subsequent introduction of *Er. hayati* (S/H) significantly reduced the progeny of *En. sophia* by 1.8 (=2.4-0.6) individuals when compared to the *En. sophia* alone treatment (S) (Table 2; *Mann-Whitney U* = 86.50, *P* < 0.0001); meanwhile *Er. hayati* produced 1.8 progeny. The increase of *Er. hayati*’s progeny (1.8) equaled the reduction of *En. sophia*’s progeny (1.8) indicating that multiparasitism on the *En. sophia* parasitized hosts was the way that *Er. hayati* interfered with *En. sophia*.

**Table 2:** Effect of interference competition of the second-introduced parasitoid female on the first-introduced parasitoid female under the limited host resource condition (Mean ± SE).

	Alone treatment		Interference treatments					
	(n = 40)		(n = 20)					
			Self		Conspecific		Heterospecific	
	S	H	S1/S1	H1/H1	S1/S2	H1/H2	S/H	H/S**^*#*^**
Progeny	2.4 ± 0.2	5.2 ± 0.4	2.1 ± 0.4	4.6 ± 0.5	1.8 ± 0.3	3.4 ± 0.3	0.6 ± 0.2	1.0 ± 0.3
Hetero. progeny	-	-	-	-	-	-	1.8 ± 0.4	1.1 ± 0.2
Fed hosts	6.2 ± 0.3	3.6 ± 0.4	7.3 ± 0.5	4.2 ± 0.6	7.4 ± 0.4	5.5 ± 0.4	6.8 ± 0.6	7.6 ± 0.5
Unpar. hosts	1.4 ± 0.2	1.2 ± 0.2	0.6 ± 0.2	1.2 ± 0.3	0.8 ± 0.3	1.1 ± 0.2	0.8 ± 0.2	0.3 ± 0.1

^#^ S, S/H, S1/S2 and S1/S1: *En. sophia* female was introduced into the leaf cage alone for 24h (with no subsequent parasitoid female introduced), followed by a heterospecific female (*Er. hayati*) for another 24h, followed by a conspecific female (*En. sophia*) for another 24h and followed by itself for another 24h, respectively; H, H/S, H1/H2 and H1/H1: *Er. hayati* was introduced into the leaf cage alone for 24h (with no subsequent parasitoid female introduced), followed by a heterospecific female (*En. sophia*) for another 24h, followed by a conspecific female (*Er. hayati*) for another 24h and followed by itself for another 24h, respectively. “Progeny” means the number of offspring of the first-introduced parasitoid female; “Hetero. progeny” means the number of offspring of the heterospecific parasitoid female which was introduced subsequently; “Fed hosts” means the number of hosts killed by parasitoids via host-feeding; “Unpar. hosts” means hosts which are neither parasitized nor host-fed by parasitoids. Numbers in parentheses indicate the sample sizes of treatments.

As compared to the *Er. hayati* alone treatment (H), the subsequent introduction of *En. sophia* (H/S) significantly reduced the progeny of *Er. hayati* by 4.2 (= 5.2 - 1.0) individuals (Table 2; *Mann-Whitney U* = 53.50, *P* < 0.0001), meanwhile it increased the number of dead hosts by 4.0 ( = 7.6 - 3.6) individuals (Table 2; *Mann-Whitney U* = 96.50, *P* < 0.0001). The equivalency between the reduction of *Er. hayati*’s progeny and the increase of dead hosts indicates that host-feeding on *Er. hayati* parasitized hosts was the way that *En. sophia* interfered with *Er. hayati*.

#### Intraspecific interference

As compared with *En. sophia* alone treatment (S), the subsequent introduction of a conspecific female (S1/S2) decreased the total number of offspring by 0.6 (Table 2, *Mann-Whitney U* = 295.00, *P* = 0.087), while the number of hosts fed increased significantly by 1.2 individuals (Table 2; *Mann-Whitney U* = 258.00, *P* = 0.024), indicating *En. sophia* could feed on the hosts parasitized by conspecific females. 

As compared with *Er. hayati* alone treatment (H), the subsequent introduction of a conspecific female (H1/H2) significantly decreased the total number of offspring by 1.8 (Table 2, *Mann-Whitney U* = 191.00, *P* = 0.0009), while the number of hosts fed increased significantly by 1.9 individuals (Table 2; *Mann-Whitney U* = 266.00, *P* = 0.034). The increase of hosts fed (1.9) equaled the reduction of *Er. hayati*’s progeny (1.8), which indicates that host-feeding on conspecific female parasitized hosts was the way that *Er. hayati* interfered with conspecific parasitoids.

#### Self-interference

The same female did not affect either the number of *En. sophia*’s progeny or of *Er. hayati*’s progeny (Table 2; *Mann-Whitney U* = 353.00 and 323.50, *P* = 0.45 and 0.22, respectively), as well as the number of hosts fed (Table 2; *Mann-Whitney U* = 278.50 and 346.50, *P* = 0.40 and 0.054, respectively). 

## Discussion

### Inter- and intraspecific interference competition

We investigated both the interspecific and intraspecific interference competition between *Er. hayati* and *En. sophia* by comparing the numbers of parasitoids progeny between alone and interference competition treatments. The results showed that 1) both species negatively affected the number of progeny of the other when introduced to one leaf cage subsequently, i.e. interspecific interference competition between *Er. hayat*i and *En. sophia* occurred when they coexisted regardless of the sequence of exposure of parasitoids and host resource richness; 2) the reduction of progeny of both *En. sophia* and *Er. hayati* in the intraspecific interference competition treatment relative to that in the alone treatment under the limited resource condition indicated intraspecific interference was present in both parasitoid species. 

These kinds of competitive interactions where there is a direct negative effect on the progeny production leading to the death of heterospecific or conspecific competitors are thought of as lethal interference competition [8,14,16]. Collier & Hunter [16] investigated the interactions between *En. sophia* and *Eretmocerus eremicus* (Rose & Zolnerowich) and proposed that multiparasitism and host-feeding on parasitized hosts are the mechanisms that these two parasitoid species interfere with each other. Both *En. sophia* and *Er. eremicus* could suppress the progeny production of the other. *Er. eremicus*’ effect on *En. sophia* appeared to reflect multiparasitism, while *En. sophia*’s effect on *Er. eremicus* appeared to reflect a combination of multiparasitism and host-feeding on parasitized hosts. At the same time, they found that both *En. sophia* and *Er. eremicus* interfered intraspecifically by host-feeding on conspecific parasitized hosts. In our study, we inferred that the effect of *En. sophia* on *Er. hayati* appeared to reflect host-feeding on parasitized hosts, while the effect of *Er. hayat*i on *En. sophia* appeared to reflect multiparasitism. Intraspecific interference of both species reflected host-feeding on conspecific parasitoid parasitized hosts. 

Host-feeding is one mechanism of either interspecific or intraspecific interference competition. According to Jervis & Kidd’s [42] study, more than 140 species belonging to 17 hymenopteran families have been observed host-feeding. Parasitoids feed on the host hemolymph to gain the nutrients needed for egg maturation [42-44]. However, if host-feeding happens on parasitized hosts, it is destructive to both the host and the parasitoid eggs or larvae within the host, resulting in interference competition [16]. In our study, no matter which of the two parasitoid species were introduced alone or sequentially, the fraction of the number of dead hosts that was induced by host-feeding was high. In particular, host-feeding was more important for *En. sophia* than *Er. hayati* as *En. sophia* host fed on far more hosts than *Er. hayat*i (see results in the alone treatment). Zang & Liu [19] also documented that *En. sophia* host-fed more than other species. The number of mature ova of 1d-old *Er. hayati* was higher than that of *En. sophia* which may contribute to the difference in nutrient need [45]. 

Multiparasitism or superparasitism is another mechanism of interference competition. These phenomena occurred when parasitoid females fail to discriminate heterospecific or conspecific parasitized host and oviposit in them [20,21,46]. Both *En. sophia* and *Er. hayati* readily conducted multiparasitism and superparasitism (Xu HY, personal observation). Since both *En. sophia* and *Er. hayati* are solitary parasitoids, the presence of more than one egg in a host can undoubtedly lead to intrinsic competition between larvae, which results in one killed through physical attack or physiological suppression [7]. 

Host availability and the order of exposure affected the outcome of interspecific competition. Under the rich host resource condition, *Er. hayati* produced the larger proportion of parasitoid progeny regardless of the order of exposure, whereas, under the limited host resource condition, the second-introduced female produced the larger proportion of progeny. One reason may account for this difference: *Er. hayati* substantially produced more progeny than *En. sophia*. The competition is relatively mild under the rich host resource condition, where *Er. hayati* have more host resources oviposition. Some argue that the second-female has an advantage: the offspring of second-ovipositing females have an apparent advantage in intrinsic competition [47]. Both *En. sophia* and *Er. eremicus* could win in multiparasitism when ovipositing secondly [16]. Both *Encarsia formosa* (Gahan) and *Encarsia luteola* (Howard) produced a greater proportion of progeny when they were introduced after each other than when they were introduced first in a competition treatment [20]. Contrary to these results, studies of interactions between *En. formosa* and *Encarsia pergandiella* (Howard) showed that *En. pergandiella* prevailed in competition, regardless of the order that the hosts were exposed to the female of these two parasitoid species [8,48]. Additionally, competition study of two oligophagous parasitoids *Sturmiopis parasitica* (Hampson) and *Cotesia sesamiae* (Cameron), which attacks the same life stages of lepidopteran cereal stemborers, showed that *S. parasitica* always outcompeted *C. sesamiae* irrespective of the order of introduction and the time interval between parasitism [17].

### Implication for host suppression

To evaluate the effectiveness of parasitoids on long term whitefly suppression, progeny production of parasitoids which represent the growth potential of the parasitoid population as well as mortality of whitefly induced by parasitoid introduction should be included in the evaluation. In the present study, mortality of *B. tabaci* MEAM1 caused by *En. sophia* and *Er. hayati* by parasitism and host-feeding in inter- and intraspecific competition treatments under different host resource conditions were studied. Results showed that the mortality of *B. tabaci* MEAM1 induced by parasitoids via parasitism, host-feeding, or both parasitism and host-feeding together varied among treatments under different host resource conditions. When *En. sophia* was introduced onto the leaf first, the subsequent introduction of itself or a conspecific female resulted in the highest total mortality of *B. tabaci* among treatments under the rich or limited host resource conditions. However, when *Er. hayati* was introduced onto the leaf first, the subsequent introduction of a heterospecific female *En. sophia* caused the highest total mortality of *B. tabaci* MEAM1 as compared to all the other treatments, under the rich or limited host resource conditions. According to the present study, the introduction of *Er. hayati* to an *En. sophia* pre-existing biological control system could achieve a higher parasitoid population, even if it seemed to be slightly inferior in suppressing *B. tabaci* as compared to a situation without subsequent introduction of *Er. hayati*; the introduction of *En. sophia* to an *Er. hayati* pre-existing biological control system could achieve a higher effect of instant control of *B. tabaci* but have lower parasitoid progeny abundance. 

In conclusion, the introduction of the exotic parasitoid *Er. hayati* to the existing parasitoid *En. sophia* could beneficially achieve higher population abundance of parasitoids providing the foundation of a sustained, effective biological system. To obtain a more comprehensive knowledge of the complicated interactions between these two parasitoids on the suppression of hosts, investigations on continuous generations should be conducted.

In general, our findings provide a theoretical foundation for application of Hymenoptera parasitoids in biological control systems. Our next goal is to investigate the complex interactions between parasitoids on continuous generations and their effect on biological control of target pest in field manipulations. 
